# Supplementary material for: Functional reconstruction of a eukaryotic-like E1/E2/(RING) E3 ubiquitylation cascade from an uncultured archaeon
Source: Nat Commun. 2017 Oct 24;8:1120. doi: 10.1038/s41467-017-01162-7 (PMC5654768; doi:10.1038/s41467-017-01162-7)
Supplement: Supplementary file 1 — Supplementary Information [file 41467_2017_1162_MOESM1_ESM.pdf]

Supplementary Information

Supplementary Notes

Supplementary Note 1.
Verification of cleavage of the *C. subterraneum* pro-ubiquitin after the C-terminal di-glycine motif
The Mascot search results identified the Ubq-GG and the Ubq-FL from the respective gel bands. Chymotrypsin was used as the digestion enzyme because it was important to identify the C-terminal peptides for the full length and cleaved proteins. If trypsin was used, it would have resulted in the formation of the C-terminal fragment TVGG from the UBQ-GG protein which would have been too small to be detected in the LC-MS/MS experiment. Conversely, chymotrypsin was used to cleave the protein to generate the VLITRTVGG peptide, which is an ideal size for detection and sequencing. The main difference between the Mascot data for the two proteins the VLITRTVGG was detected in the smaller cleaved ubiquitin band but the longer VLITRTVGGCGEPIRRAA peptide was not found. However, the C-terminal VLITRTVGGCGEPIRRAA peptide was found exclusively in the uncleaved full-length protein band, suggesting that the pro-ubiquitin was indeed cleaved at the C-terminus after the di-glycine motif by Rpn11. The functional assay displayed in Figure 1c demonstrates that only the Rpn11 cleaved ubiquitin, and not the uncleaved full-length pro-ubqiuitin control, can be adenylated and auto-ubiquitylate the E1-like and E2-like proteins.

Supplementary Note 2.
Ubiquitin and ubiquitin-like auto-modifications on archaeal E1-like homologues
In accordance with the modifications observed on the *C. subterraneum* E1 enzyme in this study, our previous investigation of the Urm1 (ubiquitin related modifier 1) modification system of the thermophilic crenarchaeon *Sulfolobus acidocaldarius* had also detected ubiquitin-like auto-modifications on the Urm1/SAMP-specific ELSA (E1-like SAMP activator) enzyme [1]. Similarly, the homologous SAMPs (small archaeal modifier proteins) have also been reported to be covalently attached to the UBA/ELSA enzyme of *Haloferax volcanii* [2], suggesting that auto-modification of E1 enzymes may be a common feature in diverse archaeal species. In order to explore the two modification sites on the *C. subterraneum* E1-enzyme further, we searched for structural homologues of CSUB_C1476 using the PHYRE2 structural prediction server [3]. This approach identified the human NEDD8-activating enzyme (NAE; PDB code: 3GZN), an E1-enzyme responsible for the activation of the ubiquitin homologue NEDD8, as the closest structural homologue of the *C. subterraneum* E1 enzyme (Figure 2a; Supplementary Table 1). Furthermore, the I-TASSER (Iterative Threading ASSEmbly Refinement) server for protein structure and function prediction [4, 5] generated a model of the *C. subterraneum* E1 enzyme, and predicted the four canonical domains observed in the eukaryotic E1 enzyme homologues: the inactive and active (ATP-binding) Rossman fold domains, the active cysteine containing domain, and a ubiquitin-like domain (UBL), respectively [6] (Figure 2a). PSI-BLAST searches also confirmed that the *C. subterraneum* C-terminal E1-like UBL domain possessed amino-acid sequence homology to the *C. subterraneum* ubiquitin homologue and several eukaryotic UBL domains (Supplementary Table 2). Closer inspection of the I-TASSER model revealed that one of the auto-modified lysine residues on the *C. subterraneum* E1-like enzyme was located within the active cysteine-containing domain (Figure 2a). This is a highly mobile domain that has been shown to undergo significant structural changes during the ubiquitylation process in eukaryotic E1-like enzymes [6]. The second modified lysine was positioned on the inactive Rossman fold domain that forms the quasi-symmetric dimer with the active Rossman fold domain, which binds the ATP required for the adenylation of the ubiquitin modifier (Figure 2a). Considering that the dynamic repositioning of the active cysteine-containing domain relative to the Rossman folds is critical for the activation of ubiquitin [6], it seems plausible that modification of either of these lysine residues might modulate the activity of the E1, perhaps performing an auto-inhibitory role.

Supplementary Note 3.
Key structural and amino-acid motif similarities between the *C. subterraneum* and eukaryotic E2 enzymes, and auto-ubiquitylation of the *C. subterraneum* E2 C-terminal tail.
The I-TASSER [7] model of the *C. subterraneum* E2 enzyme demonstrated that the first N-terminal alpha-helix harboured a motif that matched the consensus sequence observed in the equivalent helix of the eukaryotic E2 homologues (Figure 2b). This region is known to mediate interaction with the ubiquitin-binding domain of the eukaryotic E1-enzyme, and also forms the canonical binding site with E3 enzymes [8-10]. The model also revealed conservation of the essential loops 4 and 7 (including the PxxPP and D/ExWSP motifs; Figure 2b), which have been proposed to play essential roles in the specific interactions with the cognate E3 ligases in eukaryotic systems [11, 12]. In addition, there was also conservation of the loop 8 sequence motif (PNxxS)

(Figure 2b), which has been suggested to act as a substrate recognition element, as has been observed previously for the SUMO conjugating E2 [11-13].

In addition to the two specific modifications detected on the E1-enzyme, a specific ubiquitylated lysine residue was also detected on the C-terminus of the *C. subterraneum* E2 enzyme following the biochemical reconstitution of the modification reaction using the ubiquitin, E1 and E2-proteins in the presence of ATP. It seems plausible that this mono-ubiquitination may be able to influence the activity of the E2 enzyme as attachment of a ubiquitin moiety to this lysine residue on the unstructured, flexible C-terminal tail might result in the positioning of the attached ubiquitin, either in close proximity to the catalytic pocket, or alternatively to the 'backside' region of the E2 enzyme [8, 14-16]. This regulatory surface, which is positioned on the opposing face to the catalytic pocket in eukaryotic E2 enzymes, has been demonstrated to mediate non-covalent binding of ubiquitin and plays a key role in modulating the processivity of ubiquitin chain formation by the E2 enzyme [15, 17-19]. Interestingly, while PHYRE2 searches revealed that the N-terminal catalytic core ubiquitin conjugating (UBC) domain of the *Saccharomyces cerevisiae* E2 enzyme Ubc1 (PDB:1TTE [20]) displayed a predicted homology to the equivalent *C. subterraneum* UBC domain with 100% confidence, the eukaryotic homologue also possesses a C-terminal extension that consists of a flexible linker region and a ubiquitin binding domain (UBA). In the eukaryotic protein the non-covalent binding of ubiquitin to the C-terminal UBA domain allows regulation of the activity of the E2-enzyme by associating with either the catalytic pocket or the opposing 'backside' face of the enzyme [15, 16, 20, 21]. It is therefore tempting to speculate that a similar mode of regulation might be mediated in the *C. subterraneum* E2 protein following covalent modification of the C-terminal lysine residue as observed in our analysis.

Supplementary Note 4.

E1-like and E2-like phylogenies and the evolution of the operonic linkage of the archaeal ubiquitylation apparatus

The gene clusters of the putative archaeal ubiquitin-like protein modifier system identified in this study can be classified within one of three main clusters (Supplementary Figure 1). The distinction between Clusters I and II is based on E2-phylogeny, while the division between Clusters I and II and the third cluster (III) is based on E1-phylogeny presented respectively in Figure 2c. It is apparent from these phylogenetic analyses that the archaeal E1-like homologues in Clusters I and II form groupings that are distinct from other prokaryotic E1-like proteins in the ThiF/MoeB/MoeZ classes. In addition, the Cluster III E2-like archaeal homologues appear stably associated with previously reported bacterial E2-like homologues (UFBoot = 97) that are commonly associated with at least one other ubiquitin-like, E2-like, E3-like or JAB metalloprotease (DUB) component [22]. The archaeal Cluster I and II E2-like homologues appear more distantly related to these bacterial homologues, and the E1-like proteins classified within Clusters I and II most frequently harbour a C-terminal ubiquitin-like domain (Figure 1A; Supplementary Table 2B). Prior to the identification of this C-terminal ubiquitin-like domain in several archaeal E1-like enzymes [23] it was previously thought that this feature was an exclusively eukaryotic innovation. Notably, this C-terminal ubiquitin-like domain is entirely absent from the Cluster III E1-enzymes and the related bacterial homologues [24]. Furthermore, earlier bioinformatics were suggestive that the *C. subterraneum* E1-like enzyme, which has been classified within Cluster I in our current study, associates most closely with eukaryotic homologues [23]. Taken together we can summarize that the archaeal Cluster III organization appears intimately related to the bacterial clusters or operons that harbour various combinations of Ub, E1-like, E2-like, E3-RING-like and JAB peptidase homologues [24, 25]. In the bacterial systems these clusters have been demonstrated to play roles in the biogensis of metal-sulfur clusters, siderophore biosynthesis or have been suggested to function as a prokaryotic ubiquitin-like conjugation system [24, 26, 27]. A few operons containing the full complement of these ubiquitylation cascade components, including the E3-like-RING domain homologues, have also been reported in a few bacterial species such as *Frankia alni* and *Pirella staleyi* [25]. Interestingly, in these examples the E1 homologues have a conserved C-terminal extension but no defined structural domains have been identified in this region. These findings indicate that the core components of ubiquitylation systems were originally prokaryotic innovations that were initially subject to horizontal gene transfer. Diversification of the function of these key components and their subsequent genomic linkage in operons or clusters appears to coincide with the earliest appearance of *bona fide* ubiquitin modification cascades [22-25]. Our phylogenetic analyses and subsequent classification of the archaeal Cluster I, II and III groupings in this study indicates that the transition from the Cluster III to Cluster I or II arrangement likely represents a significant step towards the emergence of a more advanced archaeal system that was seemingly inherited by the earliest eukaryotic cells. In the archaeal Cluster I and II arrangement, the fusion of a ubiquitin-like domain to the C-terminus of the E1-like catalytic domain would have resulted in a more robust and stable association with the E2 homologue, and this domain arrangement was then retained during the evolution of the ubiquitylation cascade in eukaryotic cells.

Supplementary Note 5.
Commentary on the observed putative alternative ubiquitin chain linkages.
The alternative isopeptide linkages identified by mass-spectrometry on the *C. subterraneum* respective K4, K56 and K68 residues of ubiquitin moiety lead to the intriguing possibility that the archaeal ubiquitylation system could be appropriated to produce complex ubiquitin-chain signals to regulate diverse biological processes. It is well established that eukaryotic cells use a variety of ubiquitin chain conformations to regulate and potentiate a number of molecular processes. For example, while poly-K48 chain-linkages are utilised as the canonical signal to target substrates for destruction at the proteasome, K63 modifications have been commonly associated with signalling and protein sorting and trafficking at endomembrane structures. It is therefore of note that the ubiquitylated lysine residues on the *C. subterraneum* ubiquitin moiety identified in the *in vitro* biochemical reconstitution of the E1-like/E2-like/srfp(E3-like) ubiquitylation cascade occur on the surface of the modifier at regions abutting the hydrophobic patch. This positioning appears somewhat reminiscent of the distribution of the eukaryotic K48 and K63 (and K6) residues. In eukaryotic systems, K63 linked chains are commonly utilized to decorate membrane-associated cargo proteins prior to recognition by ESCRT (endosomal sorting complexes required for transport) machinery. The observed genomic linkage between components of the ubiquitylation and ESCRT apparatus in several members of the recently identified Asgard archaea [28, 29] is suggestive that a parallel ubiquitin-regulated endomembrane trafficking mechanisms might also occur in these complex eukaryotic-like archaeal relatives. Experimental verification of the involvement of putative ubiquitin linkages in endomembrane transport mechanisms in the Asgard archaea would provide valuable insights into the inheritance and functioning of these vital cellular systems during the evolution of early eukaryotic cells from complex archaeal progenitors. Indeed the development of more advanced ubiquitin signaling and the maturation of a more complex membrane trafficking apparatus may have been a pre-requisite for the acquisition of the mitochondrial endosymbiont, an event that demarcates the emergence of the first eukaryotic cell [28-30].

Supplementary Note 6.
Expansion of E3 repertoire during evolution of the eukaryotic ubiquitylation systems
Interestingly, it is thought that while the majority of eukaryotic E1 and E2 enzymes were likely derived from progenitors inherited from an archaeal source and display somewhat limited diversification during the early evolution of eukaryotes, the E3 ligases rapidly expanded into extremely broad and numerous eukaryotic-specific protein families [31]. Indeed, the E2-stimulating RING and HECT domains are often fused to a variety of additional domains with multifarious architectures that provide greater specificity to the varied modification pathways [31-36]. Thus far only examples of RING domain containing srfp have been identified in archaeal species. It will be interesting in the future to investigate new examples of both complex archaeal species and also primitive eukaryotic organisms to learn more about the evolutionary events that permitted this rapid E3-ligase expansion, which was presumably a major factor in facilitating the development of more complex cellular pathways and the emergence of the most highly-developed eukaryotic organisms.

Supplementary Note 7.
Substrate specificity of the *C. subterraneum* Rpn11 deubiquitinase (DUB) homologue.
The biochemical characterisation of the *C. subterraneum* Rpn11 in this study revealed that this DUB homologue displays a broad range of substrate specificity, acting on a wide variety of proteins including the C-terminal pro-peptide of ubiquitin, an N-terminally linked ubiquitin fusion, while also displaying deconjugating activity on all three forms of the isopeptide linkages (K4, K56, K68) detected by our mass-spectrometry analyses. These observations are consistent with the finding of Hepowit *et al* [37], who revealed that the JAMM1 zinc-dependent deubiquitylase of *Haloferax volcanii* also displayed a similar broad specificity towards a number of different substrates [37]. Indeed, in that study it was suggested that the unexpected ability of HvJAMM1 to cleave substrates with linear SAMP fusions might represent a common feature of all archaeal group 1 JAMM domain proteins [37]. Furthermore, the study proposed that, in addition to SAMP deconjugation reactions, archaeal JAMM domain metalloproteases might also be involved in the maturation of archaeal SAMP proteins by cleavage of propeptides, such as those observed in *Natrialba magadii* [38], thereby generating the diglycine motif required for conjugation events. Our analyses of the *C. subterraneum* ubiquitylation system has experimentally confirmed this ubiquitin maturation activity, as we observed that the ubiquitin homologue expressed by *C. subterraneum* can indeed be processed to a mature ubiquitin species by cleavage of the C-terminal pro-peptide to expose the di-glycine motif. It is interesting to note, however, that the phylogenetic classification of this *C. subterraneum* metalloprotease homologue places this Rpn11 homologue closely with the eukaryotic JAMM isopeptidase and DUB domains, rather than grouping with the archaeal group 1 JAMM

proteases [37, 39]. Considering the close evolutionary relationship of the *C. subterraneum* deubiquitylase to eukaryotic Rpn11 and Csn5 homologues, it is interesting that the archaeal homologue appears to display activity in the absence of any interacting partner protein or complex. By contrast, eukaryotic counterparts require incorporation within a larger multi-subunit complex (*e.g.* the regulatory 19S lid of the proteasome or the COP9 signalosome) in order to confer catalytic competency. The archaeal Rpn11 homologue therefore likely represents an interesting intermediate in the evolution of ubiquitylation systems. It will be interesting to determine if the *C. subterraneum* ubiquitylation systems do target substrates to the proteasome in accordance with the well-established eukaryotic roles. Indeed, recent studies have revealed that the ancient archaeal ubiquitin-like Urm1 and SAMP homologues of *S. acidocaldarius* and *H. volcanii* do appear to be involved in targeting substrates to the proteasome [1, 40]. It therefore seems plausible that the bona fide *C. subterraneum* ubiquitin homologue will also play a role in directing substrates to the proteasome for degradation. In this scenario it would be interesting to determine if the Rpn11 homologue forms part of a eukaryotic-like proteasomal regulatory cap complex that regulates substrate recognition and deconjugation of the ubiquitin moieties prior to processing, in a manner reminiscent of the eukaryotic UPS. Indeed, the X-ray crystal structures of an *Archaeoglobus fulgidus* JAMM homologue has proved illuminating in determining models of arrangement and activity of the JAMM isopeptidase subunits of the COP9 signalosome and the 26S proteasome macromolecular assemblies [41, 42]. The identification of these putative proteasomal regulatory cap complexes from the more complex archaeal species, such as *Aigarchaeota* and Asgard superphylum should provide valuable insights into the evolution of the sophisticated 19S proteasome lid assemblies that function in eukaryotic species.

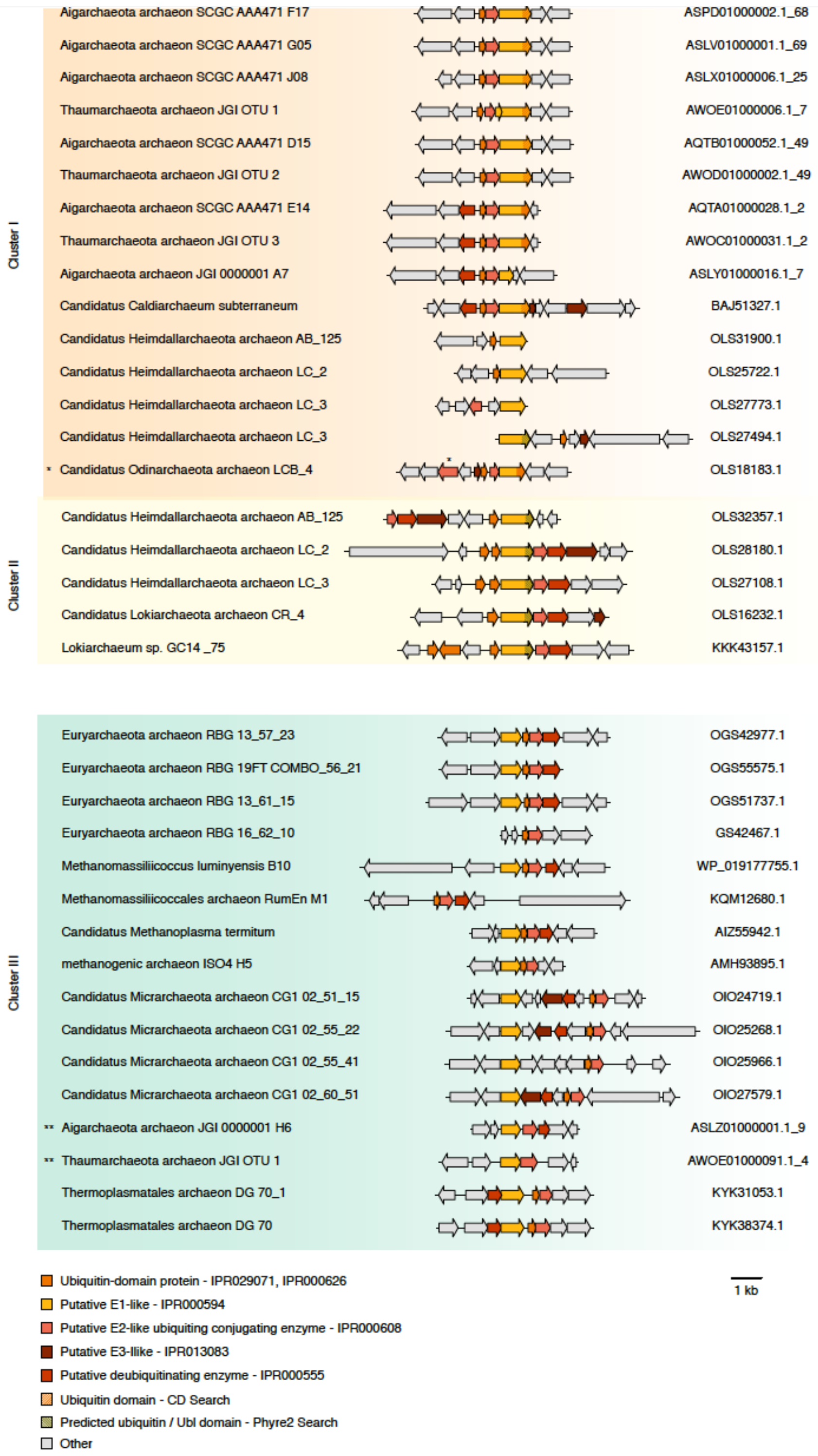

Supplementary Figure 1. Gene clusters of the putative ubiquitin-like protein modifier system identified in archaeal species: the ubiquitin, E1-like, E2-like and small RING finger protein (srfp) and Rpn11 deubiqutinase components are coloured as indicated in the key. The distinction between Clusters I and II is based on E2-phylogeny, while the division between Clusters I, II and II is based on E1-phylogeny presented respectively in Figure 2c. The bioinformatics approach used to locate and define the Clusters and also the C-terminal ubiquitin-like (UBL) domains is described in the Supplementary Methods and the evolutionary implications for the clustering is discussed in the Supplementary Notes. An additional E2-like homologue is identified in Candidatus Odinarchaeota archaeon LCB_4 is denoted by a single asterisk (also identified in the E2-like tree in Figure 2c), while the double asterisks indicate that the E2-like homologues identified in Aigarchaeota archaeon JGI 0000001 H6 and Thaumarchaeota archaeon JGI OTU 1 fall outside the main Cluster III grouping as shown in the E2-like tree in Figure 2c. Archaeal gene-clusters not belonging to cluster I, II or III are not shown (4 cases).

```
CSub1476      1 ------------------------------------MLGEVDALSRYDRQLRLEGWDQNK
Naumovozyma   1 ------------------------------------------------------------
Galdieria     1 ----------------------------MSQGKDEGKLEDLYLITLRNGPFAHESFSPSE
Columba       1 MADGEEPEKKRRRLEELLADGMAVDVGCGDTGDWDGRWNHVKKFLERSGPFTHPDFEPGT
Geospiza      1 ---------------------MAVDGGCGDSGDWEGRWNHVKKFLERSGPFTHPDFEPGT

CSub1476     25 ------LLSGRVIVAGVGALGCEVAKNLALMGVGELLLIDNDYVELSNLSRQMLYTDQDI
Naumovozyma   1 -------MDLKVLILGAGGLGCEILKNLTMMQVKEIHIVDMDTIELSNLNRQFLFSDDDI
Galdieria    33 DLLNFLWNECKVLVVGAGGLGCELLKDLALSGFRNIEVIDLDVVDVTNLNRQFLFRQQDV
Columba      61 QALDFLLSTCKVLVIGAGGLGCELLKNLALSGFRQIHVIDMDTIDVSNLNRQFLFRPKDV
Geospiza     40 QALDFLLSTCKVLVIGAGGLGCELLKNLALSGFRQIHVIDMDTIDVSNLNRQFLFRAKDV

CSub1476     79 GRPKASTAEKKIS-----LMNPLVKAKGLHTDVRKIPEETFAEADVIVSAVDNWPTRRWM
Naumovozyma  54 GKSKSITAAKYINEEHHYKKRRGVNVIPYHQDLTTFPIEFFKQFDFVISGLDSIIPRRFI
Galdieria    93 GKPKAEVAAAFIA-----KRISGINIKGHHANIYDQPREFYKQFNLVVAGLDSIDARRWL
Columba     121 GRPKAEVAAEFLN-----SRIPNCAVVAYFKKIQDMDESFYRQFHIIVCGLDSVIARRWI
Geospiza    100 GRPKAEVAAEFLN-----SRIPNCAVVPYFKKIQDMDESFYRQFHIIVCGLDSIIARRWI

CSub1476    134 NSMAVHVGT-------------PLVDVATDGYYGNVQTVIPGVTSCLECHAEALIPSDIQ
Naumovozyma 114 NEKLIEITR--ETGF---ETCIPLIDGGTEGFKGHVKTIIPGITACWECSIDTLPTSQDT
Galdieria   148 NETLIDLVETNDDGTIDVSTVIPLIDGGTEGFRGQARVIIPKMSACFECNLDLFPP-QIS
Columba     176 NGMLMSFLH-YEDGVLDPSSIIPLIDGGTEGFKGNVRVIIPGMTACVECTLELYPP-QVN
Geospiza    155 NGMLMSFLH-YEDGVLDPSSIIPLIDGGTEGFKGNVRVIIPGMTACVECTLALYPP-QVN

CSub1476    181 ASECSLRR--RTPNDLVK--------DLSERGISINLSDAETLFQHNIKTVYDIKFAPQT
Naumovozyma 169 VPMCTIANNPRSLEHIIEYVISKRSENEMEEGQKGEIEESS---EVVIDTILKKCYERAR
Galdieria   207 YPLCTIANTPRLPEHCIEYASVILWPQQQPFGAGTKVDGDN---PEHVKWIFERAQERAN
Columba     234 FPMCTIASMPRLPEHCIEYVRILQWPKEQPFGEGVALDGDD---PEHIQWIYQKSLERAS
Geospiza    213 FPMCTIASMPRLPEHCIEYVRILQWPKEQPFGEGIALDGDD---PEHIQWIYQKSLERAS

CSub1476    231 VLDQMDKSLREQVIQLRSLLNPKMPALQSISATVSGLASFEVVRLLHKGSLGRSLN---G
Naumovozyma 226 MFNID--TIRLNKEYLLGILKEIIPAVSSTNAMIAAACCNEMLRIYS--DM-IDLNEDGN
Galdieria   264 QFHIQGVTYRLS----QGVIKHIIPAVASTNAIVAASCANEAFKLAT--YIANPLN---N
Columba     291 QFNIKGVTYRLT----QGVVKRIIPAVASTNAAIAAVCATEVFKIAT--SAYIPLN---N
Geospiza    270 QFNIKGVTYRLT----QGVIKRIIPAVASTNAVIAAVCATEVFKIAT--SAYVPLN---N

CSub1476    288 MMVFDGLRGRLS-RIKLERNVNCHVCGYSEDKPVQINVAPNETIADLRERI---------
Naumovozyma 281 FTIINGAEGCFTYTFSYDRRPDCLVCG------------------DLFQ-----------
Galdieria   315 YMLYNGESGVYTYAFETERREECPACG--RAQPKKICVSPKWTLADLIEVLREDTELRVK
Columba     342 YLVFNDVDGLYTYTFEAERKENCPACS---QLPQNIEISPSAKLQEILDYLTNNASLQMK
Geospiza    321 YLVFNDVDGLYTYSFEAERKENCPACS---QLPQNIEISPSAKLQEILDYLTNNASLQMK
```

Supplementary Figure 2. Multiple sequence alignments of the *C. subterraneum* E1-like protein amino-acid sequence (Csub1476) with the closest eukaryotic E1-ubiquitin-activating homologues (*Naumovosyma dairenensis* [yeast], *Galdieria sulphuraria* [extremophilic red alga], *Columba livia* [bird], *Geospiza fortis* [bird]). The glycine-rich ATP-binding motif (shown in blue in Figure 2a) is highlighted by the blue bar. Yellow circles denote the zinc-binding and catalytic cysteine residues. Orange circles indicated the conserved residues involved in ATP hydrolysis indicated in Figure 2a as identified in [24].

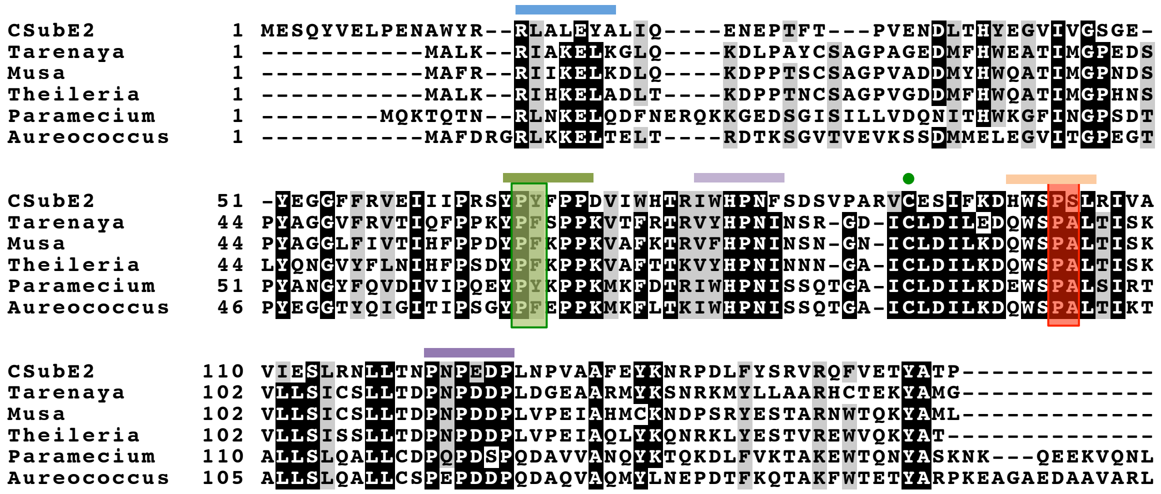

Supplementary Figure 3. Multiple sequence alignments of the *C. subterraneum* E2-like protein amino-acid sequence (Csub1476) with the closest eukaryotic E2-ubiquitin-conjugating homologues (*Paramecium tetraurelia* [Alveolata ciliate], *Musa acuminata* [banana], *Aureococcus anophagefferens* [alga], *Tarenaya hassleriana* [spider flower], *Theileria orientalis* [Apicomplexan parasite]). The blue highlights the E1/E3 interacting alpha-helix indicated in Figure 2b. Green, light purple, orange and dark purple bars highlight the amino-acid residues conserved on the E2 conserved loop 4, the HPN loop, loop 7, and loop 8, respectively. The green circle denotes the catalytic cysteine. The conserved PY or PS/A motifs involved in E3 ligase-RING domain interaction are highlighted with green and red boxes on loops 4 and 7, respectively.

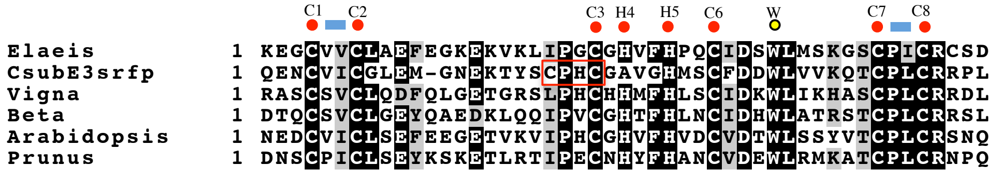

Supplementary Figure 4. Multiple sequence alignments of the *C. subterraneum* E3-H2-RING- like protein amino-acid sequence (Csub1476) with the closest eukaryotic E3-RING homologues (*Elaeis guineensis* [oil-palm], *Vigna radiata* [mung bean], *Beta vulgaris* [sugar beet], *Arabidopsis lyrata* [rockcress], *Prunus persica* [peach]). H2-RING family proteins display the consensus: C1-X(2)-C2-X(9-27)-H3-X(1-3)-C4-X(2)-H5-X(2)-C6-X(4-48)-C7-(X2)-C8. These zinc-binding cysteine and histidine residues are highlighted by red circles. Note that the position of the *C. subterraneum* C3 and H4 residues are out of alignment by 2 residues (red box). The conserved tryptophan residue (yellow circle) and hydrophobic interaction interfaces between cysteines C1 and C2 and C7 and C8 (blue bars) are also highlighted.

A

	C-score	Estimated TM-score	Estimated RMSD
***C. subterraneum* E1-like**	**-0.34**	**0.67±0.13**	**7.5±4.3Å**
***C. subterraneum* E2-like**	**0.05**	**0.72±0.11**	**4.9±3.2Å**
***C. subterraneum* E3-like**	**-0.12**	**0.70±0.12**	**3.6±2.5Å**

Bi

C. subterraneum E1-like	PDB	Confidence	% Identity	Organism	Description
1	3kyC	100	23	*H. sapiens*	sumo-activating enzyme subunit 2
2	1y8q	100	23	*H. sapiens*	ubiquitin-like 2 activating enzyme e1b
3	3kyd	100	24	*H. sapiens*	sumo-activating enzyme subunit 2
4	3gzn	100	25	*H. sapiens*	nedd8-activating enzyme e1 catalytic subunit
5	2nvuB	100	27	*H. sapiens*	maltose binding protein/nedd8-activating enzyme

Bii

C. subterraneum E2-like	PDB	Confidence	% Identity	Organism	Description
1	4ddi	100	33	*H. sapiens*	ubiquitin-conjugating enzyme e2 (ubch5b)
2	1yla	100	34	*H. sapiens*	ubiquitin-conjugating enzyme e2
3	3e 46	100	34	*H. sapiens*	ubiquitin-conjugating enzyme e2
4	3bzh	100	34	*H. sapiens*	ubiquitin-conjugating enzyme e2
5	1y6l	100	35	*H. sapiens*	ubiquitin-conjugating enzyme e2
6	1zdn	100	29	*H. sapiens*	UBC-like
7	4gpr	100	35	*E. histolytica*	ubiquitin-conjugating enzyme
8	1i7k	100	26	*H. sapiens*	ubiquitin-conjugating enzyme e2 (UBCH10)
9	1i7k	100	26	*H. sapiens*	ubiquitin-conjugating enzyme e2 (UBCH10)
10	1qcq	100	37	*S. cerevisiae*	UBC-like (yeast Ubc4)
11	1j7d	100	34	*H. sapiens*	UBC-like (hUbc13)
12	1tte	100	30	*S. cerevisiae*	ubiquitin-conjugating enzyme e2 (Ubc1)

Biii

C. subterraneum E3-like	PDB	Confidence	% Identity	Organism	Description
1	1x4j	99.8	26	*H. sapiens*	ring finger protein 38
2	2l0b	99.8	22	*H. sapiens*	E3 ubiquitin-protein ligase praja-1
3	5d0k	99.8	26	*H. sapiens*	ring finger protein 165 (RNF165)
4	2ep4	99.8	25	*H. sapiens*	nedd8-activating enzyme e1 catalytic subunit
5	2ect	99.8	22	*H. sapiens*	ring finger protein 126 (C3HC4 type (RING finger))

Supplementary Table 1. Summary of the modelling for the *C. subterraneum* E1-like, E2-like and srfp (E3-like) ubiquitylation enzymes. (a) Estimated accuracy of the I-TASSER [4, 7] generated models displayed in Figures 2 and 3. The C-score is a confidence score typically in the range -5 to +2, where a higher value indicates a model with higher confidence. The estimated Template Modelling (TM)-score is a metric for assessing the structural similarity between two-models and has a value of 0 to 1, where a perfect match is indicated by a score of 1. Scores higher than 0.5 are generally indicative of a similar fold. The estimated RMSD (root mean square deviation) measures the standard deviation between the spatial distance between pairs of equivalent atoms in the observed structure and the predicted model. (b) PHYRE2 (Protein Homology/analogY Recognition Engine V 2.0) searches predicting eukaryotic structural homologues of the *C. subterraneum* (Bi) E1-like (Bii) E2-like and (Biii) srfp (E3-like) with 99.8-100% confidence (this is a confidence score that is representative of the probability (ranging from 0 to 100%) that the matching sequences between the input and template is a true homology). Searches were performed on the PHYRE2 protein fold recognition server [3]. (http://www.sbg.bio.ic.ac.uk/phyre2/html/page.cgi?id=index).

A

Protein homologue [Organism]	Max Score	Total Score	Query Cover	E Value	Ident	Acccession
ubiquitin-like protein [Candidatus Caldiarchaeum subterraneum]	42	42	55%	0.006	35%	BAJ48537.1
Chain A, Solution Structure Of Ubiquitin-like Protein From Caldiarchaeum Subterraneum	42	42	55%	0.006	35%	2MQJ_A
hypothetical protein TRIADDRAFT_29688 [Trichoplax adhaerens]	39.7	39.7	94%	0.22	27%	XP_002115524.1
UV excision repair protein Rad23 [Kwoniella mangroviensis CBS 10435]	38.9	38.9	73%	0.38	31%	OCF58560.1
UV excision repair protein Rad23 [Kwoniella mangroviensis CBS 8886]	38.9	38.9	73%	0.44	31%	OCF78567.1
hypothetical protein RMCBS344292_01397 [Rhizopus microsporus]	38.5	38.5	81%	0.47	34%	CEI86975.1
UV excision repair protein Rad23 [Kwoniella mangroviensis CBS 8507]	38.5	38.5	73%	0.51	31%	XP_019000751.1
UV excision repair protein Rad23 [Dacryopinax primogenitus]	38.1	38.1	77%	0.7	29%	EJU04294.1
hypothetical protein RMATCC62417_18576 [Rhizopus microsporus]	37.7	37.7	81%	0.76	34%	CEG84824.1
hypothetical protein HMPREF1544_11274 [Mucor circinelloides f. circinelloides 1006PhL]	37.7	37.7	53%	1	40%	EPB81995.1
nitrobenzoate reductase [Alcanivorax sp. P2S70]	37.7	37.7	61%	1	39%	WP_022985072.1
hypothetical protein MOQ_003119 [Trypanosoma cruzi marinkellei]	37.7	37.7	91%	1.1	28%	EKF33025.1
ubiquitin [Theileria orientalis strain Shintoku]	37	37	60%	1.3	31%	XP_009690160.1

B

Organism	Accession	Rank	PDB	Model Organism	Confidence	% identity	Description
Candidatus Heimdallarchaeota archaeon LC_3	OLS27494	2	2nvu	*Homo sapiens*	37.42%	17%	nedd8-activating enzyme
Candidatus Heimdallarchaeota archaeon AB_125	OLS32357	1	3kyd	*Homo sapiens*	95.45%	24%	sumo-activating enzyme subunit 2
Candidatus Heimdallarchaeota archaeon LC_2	OLS28180.1	1	2kzr	*Mus musculus*	94.41%	23%	solution nmr structure of ubiquitin thioesterase otu1
		2	2n7d	*Homo sapiens*	91.15%	17%	solution structure of the ubl domain of human ddi2
		3	1we6	*Arabidopsis thaliana*	89.32%	18%	beta-Grasp (ubiquitin-like)
Candidatus Heimdallarchaeota archaeon LC_3	OLS27108.1	1	2nvu	*Homo sapiens*	97.49%	19%	nedd8-activating enzyme
Candidatus Lokiarchaeota archaeon CR_4	OLS16232	16	3kyc	*Homo sapiens*	10.10%	17%	sumo-activating enzyme subunit 2
Lokiarchaeum sp. GC14 _75	KKK43157_1	1	3gzn	*Homo sapiens*	84.31%	17%	nedd8-activating enzyme e1 catalytic subunit

Supplementary Table 2. C-terminal ubiquitin-like (UBL) domains identified in *C. subterraneum* and Asgard E1-like homologues (also see Figure 1A). (a) PSI-BLAST analysis of the C-terminal region of the *C. subterraneum* E1-like (Csub_C1476) protein identifies a UBL domain with amino-acid sequence homology to *C. subterraneum* ubiquitin and homologous eukaryotic UBL domains. (b) β–grasp fold UBL domains predicted by PHYRE2 searches of the C-terminal domains of the Cluster I and Cluster II *Heimdallarcheota* and *Lokiarcheota* E1 homologues. Searches were performed on the PHYRE2 protein fold recognition server Searches were performed on the PHYRE2 protein fold recognition server [3].

Oligonucleotide name	Sequence (5' to 3')
CSUB_1474UBIforNdeI	GCGCATATGAAGATTAAGATTGTTCCCGCTGTCGGAGGAGGTTCACCCTTGGAGCTTGAGGTTGCTCC
CSUB_1474UBIrevXhoIFL	GCGCTCGAGTCAGGCAGCTCGACGTATTGGCTCTCCACATCCACCCACC
CSUB_1474UBIrevXhoIGG	GCGCTCGAGTCATCCACCCACCGTCCGCGTGATGAGTACGAATTTGTCTCCATC
CSUB1474_UBIrevNdeIGG	GCGCATATGTCCACCCACCGTCCGCGTGATGAGTACGAATTTGTCTCCATC
CSUB_C1476E1NdeIfor	GCGCATATGTTGGGTGAGGTTGACGCGCTGTCGAGGTACGATCGCCAGCTACGGCTTGAGGG
CSUB_C1476E1XhoIrev	GCGCTCGAGCTAATCACCAGCCTCAATCAGCTTCACCTTCACAGCCACCGGTGTTGCTCTCCTGCTTGAATGGATATAAAGTATGTCTCCATCACAAATGCCTGCCG
CSUB_C1475E2NdeiFor	GCGCATATGGAGAGCCAATACGTCGAGCTGCCTGAAAACGCGTGGTACAGGCGGCTGGCTCTTGAGTATGC
CSUB_C1475E2Xhoirev	GCGCTCGAGCTACAAACCCTTCCAACGCTTCTTTCCGAAAGCCTGCTCAGGCGTAGCGTATGTTTCG
CSUB_C1477srfpE3Ndeifor	GCGCATATGAGGCTGGTGATTAGAGAGGTCAACGC
CSUB_C1477srfpE3Xhoirev	GCGCTCGAGTCACATCTCTACCAGCGGCCTGCGGCAAAGCGGGC
CSUB1473srfpp1NdeIfor	GCGCATATGCGTGTAAGAATCTATCCGCTGGCCTTGGCG
CSUB1473srfpp1XhoIrev	GCGCTCGAGAACCTTTCCTGCCGACTTGCCGACCAGCAAGCC
CSUB1473srfpp2XhoIfor	GCGCTCGAGATATGGGATGCTGTCACGGGTGAGC
CSUB1473srfpp2XhoIREV	GCGCTCGAGTTATGCTCCGAATAGCTTTTTAGCTTTTCC
SaciMre11forNdeI	GCGCATATGCAATTACTTCATATTTCAGACACTCATCTAGG
SaciMre11revXhoI	GCGCTCGAGTCATTTGTCCTCAACACCTGCAAATTTTTTCAAAAG
SaciRad50forNdeI	GCGCATATGATAATCAGAGAGATAAGATTACAAAACTTCCTTAG
SaciRad50revXhoI	GCGCTCGAGTTATCTATCATAACTTGACACCTCTACCTTACTAGTG
CSUBMUTUBQT46D	GCCACCCGACACCACCCGCCTA*GAC*TACAAGGGTAGAGCCCTCAAAGACACCGAAAC
CSUBMUTE2PY6768AAmutLoop4	GTAGAAATAATCATTCCACGGTCTTAC*GCAGCT*TTCCCACCCGATGTTATTTGGCACACACGG
CSUBMUTE2PS103104AAmutLoop7	GTGAATCAATTTTCAAAGACCACTGGTCA*GCCGCT*CTCCGCATAGTGGCCGTAATCGAGTC
CSUBMUTE2PS103104AQmutLoop7	GTGAATCAATTTTCAAAGACCACTGGTCA*GCCCAA*CTCCGCATAGTGGCCGTAATCGAGTC
CsubE3mutW58A	GTTGGTCACATGTCGTGTTTCGACGAC*GCG*CTGGTGGTGAAGCAGACGTGCCCGCTTTGCCG
CsubE3mutI30Q	CCGAAGCCAAGGCCGAAGCAAGAGAACTGTGTA*CAA*TGTGGCCTGGAGATGGGGAATG
CSubE3R69A	GGCTGGTGGTGAAGCAGACGTGCCCGCTTTGC*GCC*AGGCCGCTGGTAGAGATGTGACTCGAGCAC

Supplementary Table 3. Oligonucleotides used in the study. The *Nde*I or *Xho*I restriction sites used for cloning into the pET28a expression vector (Novagen) are underlined. The *CSUB_C1473* was amplified and cloned as separate N-terminal and C-terminal fragments due to an *Xho*I restriction site within the *CSUB_C1473*ORF. The ubiquitin-GG ORF was also amplified with *Nde*I sites at both termini and cloned at the *Nde*I site at the start of a superfolder-GFP ORF previously cloned into a pET30 expression vector [19], thereby generating an N-terminal ubiquitin:GFP fusion. Primers used for mutagenesis (QuikChange, Agilent) are presented at the bottom of the table, with the mutated regions italicized and underlined

Supplementary References

1. *Anjum, R.S., et al., Involvement of a eukaryotic-like ubiquitin-related modifier in the proteasome pathway of the archaeon Sulfolobus acidocaldarius. Nat Commun, 2015.* ***6****: p. 8163.*
2. *Humbard, M.A., et al., Ubiquitin-like small archaeal modifier proteins (SAMPs) in Haloferax volcanii. Nature, 2010.* ***463****(7277): p. 54-60.*
3. *Kelley, L.A., et al., The Phyre2 web portal for protein modeling, prediction and analysis. Nat Protoc, 2015.* ***10****(6): p. 845-58.*
4. *Zhang, Y., I-TASSER server for protein 3D structure prediction. BMC Bioinformatics, 2008.* ***9****: p. 40.*
5. *Roy, A., A. Kucukural, and Y. Zhang, I-TASSER: a unified platform for automated protein structure and function prediction. Nat Protoc, 2010.* ***5****(4): p. 725-38.*
6. *Lorenz, S., et al., Macromolecular juggling by ubiquitylation enzymes. BMC Biol, 2013.* ***11****: p. 65.*
7. *Yang, J., et al., The I-TASSER Suite: protein structure and function prediction. Nat Methods, 2015.* ***12****(1): p. 7-8.*
8. *Stewart, M.D., et al., E2 enzymes: more than just middle men. Cell Res, 2016.* ***26****(4): p. 423-40.*
9. *Metzger, M.B., et al., RING-type E3 ligases: master manipulators of E2 ubiquitin-conjugating enzymes and ubiquitination. Biochim Biophys Acta, 2014.* ***1843****(1): p. 47-60.*
10. *Lee, I. and H. Schindelin, Structural insights into E1-catalyzed ubiquitin activation and transfer to conjugating enzymes. Cell, 2008.* ***134****(2): p. 268-78.*
11. *Winn, P.J., et al., Determinants of functionality in the ubiquitin conjugating enzyme family. Structure, 2004.* ***12****(9): p. 1563-74.*
12. *Papaleo, E., et al., Loop 7 of E2 enzymes: an ancestral conserved functional motif involved in the E2-mediated steps of the ubiquitination cascade. PLoS One, 2012.* ***7****(7): p. e40786.*
13. *Bernier-Villamor, V., et al., Structural basis for E2-mediated SUMO conjugation revealed by a complex between ubiquitin-conjugating enzyme Ubc9 and RanGAP1. Cell, 2002.* ***108****(3): p. 345-56.*
14. *Berndsen, C.E. and C. Wolberger, New insights into ubiquitin E3 ligase mechanism. Nat Struct Mol Biol, 2014.* ***21****(4): p. 301-7.*
15. *Brzovic, P.S., et al., A UbcH5/ubiquitin noncovalent complex is required for processive BRCA1-directed ubiquitination. Mol Cell, 2006.* ***21****(6): p. 873-80.*
16. *Buetow, L., et al., Activation of a primed RING E3-E2-ubiquitin complex by non-covalent ubiquitin. Mol Cell, 2015.* ***58****(2): p. 297-310.*
17. *Ranaweera, R.S. and X. Yang, Auto-ubiquitination of Mdm2 enhances its substrate ubiquitin ligase activity. J Biol Chem, 2013.* ***288****(26): p. 18939-46.*
18. *Page, R.C., et al., Structural insights into the conformation and oligomerization of E2~ubiquitin conjugates. Biochemistry, 2012.* ***51****(20): p. 4175-87.*
19. *Sakata, E., et al., Crystal structure of UbcH5b~ubiquitin intermediate: insight into the formation of the self-assembled E2~Ub conjugates. Structure, 2010.* ***18****(1): p. 138-47.*
20. *Merkley, N. and G.S. Shaw, Solution structure of the flexible class II ubiquitin-conjugating enzyme Ubc1 provides insights for polyubiquitin chain assembly. J Biol Chem, 2004.* ***279****(45): p. 47139-47.*
21. *Ye, Y. and M. Rape, Building ubiquitin chains: E2 enzymes at work. Nat Rev Mol Cell Biol, 2009.* ***10****(11): p. 755-64.*
22. *Burroughs, A.M., L.M. Iyer, and L. Aravind, The natural history of ubiquitin and ubiquitin-related domains. Front Biosci (Landmark Ed), 2012.* ***17****: p. 1433-60.*
23. *Koonin, E.V. and N. Yutin, The dispersed archaeal eukaryome and the complex archaeal ancestor of eukaryotes. Cold Spring Harb Perspect Biol, 2014.* ***6****(4): p. a016188.*
24. *Burroughs, A.M., L.M. Iyer, and L. Aravind, Natural history of the E1-like superfamily: implication for adenylation, sulfur transfer, and ubiquitin conjugation. Proteins, 2009.* ***75****(4): p. 895-910.*
25. *Burroughs, A.M., L.M. Iyer, and L. Aravind, Functional diversification of the RING finger and other binuclear treble clef domains in prokaryotes and the early evolution of the ubiquitin system. Mol Biosyst, 2011.* ***7****(7): p. 2261-77.*
26. *Iyer, L.M., A.M. Burroughs, and L. Aravind, The prokaryotic antecedents of the ubiquitin-signaling system and the early evolution of ubiquitin-like beta-grasp domains. Genome Biol, 2006.* ***7****(7): p. R60.*
27. *Godert, A.M., et al., Biosynthesis of the thioquinolobactin siderophore: an interesting variation on sulfur transfer. J Bacteriol, 2007.* ***189****(7): p. 2941-4.*
28. *Spang, A., et al., Complex archaea that bridge the gap between prokaryotes and eukaryotes. Nature, 2015.* ***521****(7551): p. 173-9.*
29. *Zaremba-Niedzwiedzka, K., et al., Asgard archaea illuminate the origin of eukaryotic cellular complexity. Nature, 2017.* ***541****(7637): p. 353-358.*

30. *Koonin, E.V., The origin of introns and their role in eukaryogenesis: a compromise solution to the introns-early versus introns-late debate? Biol Direct, 2006.* ***1****: p. 22.*
31. *Zuin, A., M. Isasa, and B. Crosas, Ubiquitin signaling: extreme conservation as a source of diversity. Cells, 2014.* ***3****(3): p. 690-701.*
32. *Grau-Bove, X., A. Sebe-Pedros, and I. Ruiz-Trillo, The eukaryotic ancestor had a complex ubiquitin signaling system of archaeal origin. Mol Biol Evol, 2015.* ***32****(3): p. 726-39.*
33. *Ardley, H.C. and P.A. Robinson, E3 ubiquitin ligases. Essays Biochem, 2005.* ***41****: p. 15-30.*
34. *Rotin, D. and S. Kumar, Physiological functions of the HECT family of ubiquitin ligases. Nat Rev Mol Cell Biol, 2009.* ***10****(6): p. 398-409.*
35. *Spratt, D.E., H. Walden, and G.S. Shaw, RBR E3 ubiquitin ligases: new structures, new insights, new questions. Biochem J, 2014.* ***458****(3): p. 421-37.*
36. *Deshaies, R.J. and C.A. Joazeiro, RING domain E3 ubiquitin ligases. Annu Rev Biochem, 2009.* ***78****: p. 399-434.*
37. *Hepowit, N.L., et al., Archaeal JAB1/MPN/MOV34 metalloenzyme (HvJAMM1) cleaves ubiquitin-like small archaeal modifier proteins (SAMPs) from protein-conjugates. Mol Microbiol, 2012.*
38. *Siddaramappa, S., et al., A comparative genomics perspective on the genetic content of the alkaliphilic haloarchaeon Natrialba magadii ATCC 43099T. BMC Genomics, 2012.* ***13****: p. 165.*
39. *Nunoura, T., et al., Insights into the evolution of Archaea and eukaryotic protein modifier systems revealed by the genome of a novel archaeal group. Nucleic Acids Res, 2011.* ***39****(8): p. 3204-23.*
40. *Fu, X., et al., Ubiquitin-Like Proteasome System Represents a Eukaryotic-Like Pathway for Targeted Proteolysis in Archaea. MBio, 2016.* ***7****(3).*
41. *Tran, H.J., et al., Structure of the Jab1/MPN domain and its implications for proteasome function. Biochemistry, 2003.* ***42****(39): p. 11460-5.*
42. *Ambroggio, X.I., D.C. Rees, and R.J. Deshaies, JAMM: a metalloprotease-like zinc site in the proteasome and signalosome. PLoS Biol, 2004.* ***2****(1): p. E2.*
